# A spatial vaccination strategy to reduce the risk of vaccine-resistant variants

**DOI:** 10.1371/journal.pcbi.1010391

**Published:** 2022-08-10

**Authors:** Xiyun Zhang, Gabriela Lobinska, Michal Feldman, Eddie Dekel, Martin A. Nowak, Yitzhak Pilpel, Yonatan Pauzner, Baruch Barzel, Ady Pauzner

**Affiliations:** 1 Department of Physics, Jinan University, Guangzhou, China; 2 Department of Molecular Genetics, Weizmann Institute of Science, Israel; 3 School of Computer Science and Center for Combating Pandemics, Tel Aviv University, Israel; 4 Department of Economics, Northwestern University, Illinois, United States of America, and School of Economics, Tel Aviv University, Israel; 5 Department of Mathematics and Department of Organismic and Evolutionary Biology, Harvard University, Massachusetts, United States of America; 6 Hayovel Elementary School, Tel-Aviv, Israel; 7 Department of Mathematics and Gonda Multidisciplinary Brain Research Center Bar-Ilan University, Israel, and Network Science Institute, Northeastern University, Boston, Massachusetts, United States of America; 8 School of Economics and Center for Combating Pandemics, Tel Aviv University, Israel; Fundação Getúlio Vargas: Fundacao Getulio Vargas, BRAZIL

## Abstract

The COVID-19 pandemic demonstrated that the process of global vaccination against a novel virus can be a prolonged one. Social distancing measures, that are initially adopted to control the pandemic, are gradually relaxed as vaccination progresses and population immunity increases. The result is a prolonged period of high disease prevalence combined with a fitness advantage for vaccine-resistant variants, which together lead to a considerably increased probability for vaccine escape. A spatial vaccination strategy is proposed that has the potential to dramatically reduce this risk. Rather than dispersing the vaccination effort evenly throughout a country, distinct geographic regions of the country are sequentially vaccinated, quickly bringing each to effective herd immunity. Regions with high vaccination rates will then have low infection rates and vice versa. Since people primarily interact within their own region, spatial vaccination reduces the number of encounters between infected individuals (the source of mutations) and vaccinated individuals (who facilitate the spread of vaccine-resistant strains). Thus, spatial vaccination may help mitigate the global risk of vaccine-resistant variants.

## Author summary

The COVID-19 pandemic demonstrated that the process of global vaccination against a novel virus can be a prolonged one. During the period of vaccination, the level of infection remains high, and each infection has a small chance to mutate into a vaccine-resistant variant. Moreover, the fact that large numbers of people are being vaccinated generates an evolutionary advantage to vaccine resistance, such that even one infection with a resistant variant will likely spread within the population and become the dominant variant. Thus, a prolonged vaccination campaign can result in vaccine escape. To reduce this risk, we propose a spatial vaccination strategy. Rather than dispersing the vaccination effort evenly throughout a country, distinct geographic regions of the country are sequentially vaccinated, quickly bringing each to effective herd immunity. Regions with high vaccination rates will then have low infection rates and vice versa. Since people primarily interact within their own region, spatial vaccination reduces the number of encounters between infected individuals (the source of mutations) and vaccinated individuals (who facilitate the spread of vaccine-resistant strains). Thus, even if a global vaccination campaign requires a prolonged period of time, the risk of vaccine escape is reduced when using a spatial strategy.

## 1. Introduction

A prime goal of vaccination during an ongoing pandemic is the rapid attainment of herd immunity, a state in which the proportion of immunized individuals is large enough to block the spread of the virus. The literature has focused on optimization strategies for efficient vaccination campaigns of large populations during a pandemic. These strategies are often designed to exploit the structure of social networks, based on the idea that the transmission dynamics are strongly intertwined with the network’s intrinsic connectivity patterns [1]. Thus, for example, network heterogeneity motivates the prioritized vaccination of “super-spreaders” [2]. At the mesoscopic scale, it was found that pandemic intervention strategies that target local network structures significantly outperform those that solely focus on the entire network structure simultaneously [3].

In addition to rapid eradication of the current pathogenic strain, an important aim of a vaccination campaign should be to minimize the chance of emergence, due to mutation, of a next strain, and in particular a vaccine-resistant strain that may undermine the entire campaign [4–17]. Indeed, if a vaccine-resistant variant appears by spontaneous mutation during a vaccination campaign it may have a clear advantage over the original strain, against which vaccines were targeted, since it can infect both vaccinated and unvaccinated individuals. Recent mathematical modeling has, in fact, shown that averting such escape scenarios is only possible under a combination of rapid vaccination and strict social distancing [18], a situation which the current campaign has shown to be unfeasible.

Given the relatively slow pace of vaccination, is it possible to mitigate the risk that vaccine resistance will emerge? The solution proposed here is based on spatial vaccination, a new vaccination strategy that has the potential to dramatically reduce the probability of this undesired evolutionary development. We focus on the current COVID-19 pandemic as a case study and in particular on the period preceding the appearance of the highly contagious though less severe Omicron variant.

**Fig 1:**
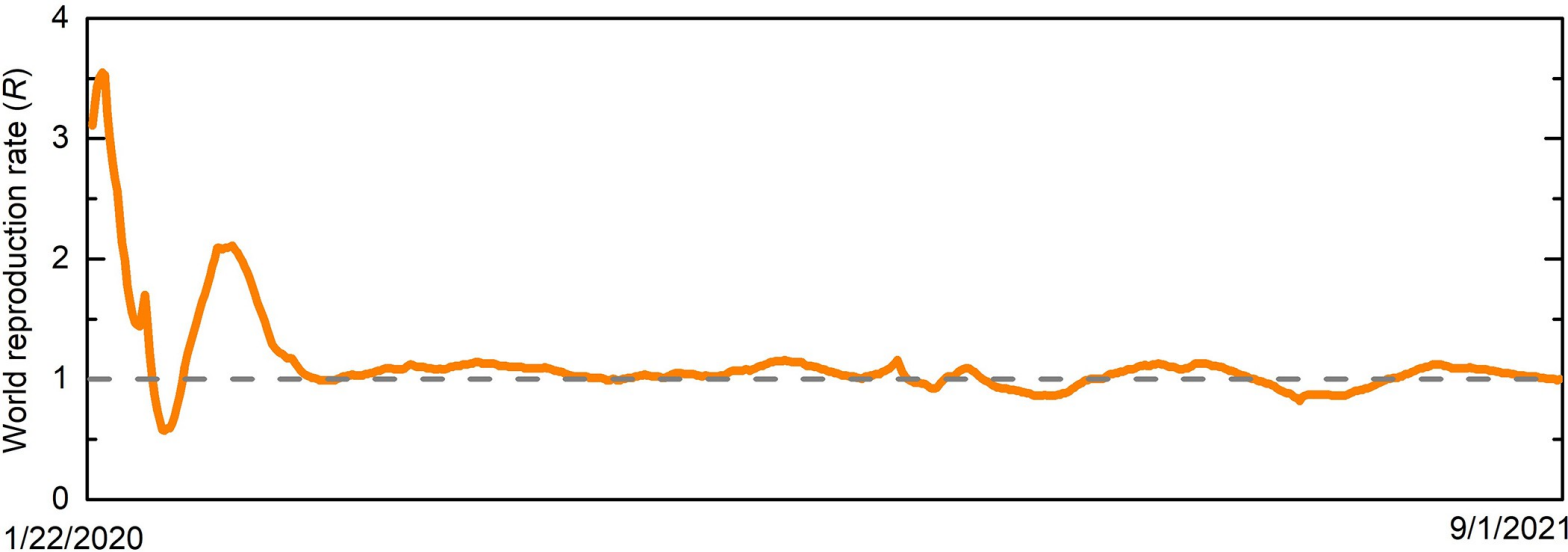
The global reproduction rate (R) during the period 2/20 to 8/21 [20,21]. As a result of adaptive social distancing, society converges to a state in which R is maintained in the vicinity of unity (dashed line). It can therefore be expected that as vaccination progresses and population immunity is gradually acquired, social distancing practices will be relaxed, thus maintaining R at about 1.

Since the initial COVID-19 strains (namely, Wuhan and Alpha) were relatively severe, and since no vaccine existed at the time, strict social distancing measures were employed to keep the pandemic under control and prevent it from proliferating. These measures were both imposed by the authorities and also driven by individuals’ independent response to the spread. The result was an ongoing adaptive behavior that reacted to the severity of the spread, thus maintaining the effective reproduction number R at around unity (Fig 1) [19]. In particular, social distancing measures were gradually relaxed as vaccination progressed and population immunity increased. Such a combination of vaccination and adaptive social distancing may have crucial implications for vaccine escape. Indeed, we depart from the canonical SIR models and explicitly take into account adaptive social distancing.

**Fig 2:**
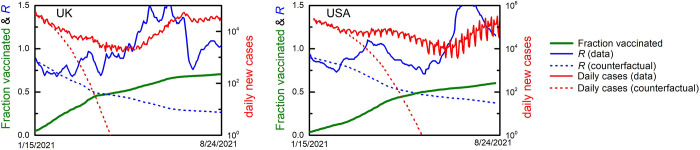
Actual vs. predicted pandemic status in the UK and US during the vaccination campaign. As the fraction of the (first-dose) vaccinated (green line) increases, the extrapolated R (dotted blue line) declines. This extrapolation assumes that factors such as the social distancing restrictions, variant composition, weather, etc. remain unchanged from the start of the vaccination campaign. The empirically measured R (solid blue line) has remained in the vicinity of unity (with a temporary jump to 1.5 just after the introduction of the more infectious delta variant). Furthermore, the extrapolated number of infections (dotted red line) declines much faster than the actual number (solid red line). These trends indeed confirm that adaptive social behavior leads to a relaxation in prophylactic measures, in response to the accumulation of population immunity.

To understand the effect of adaptive social distancing more clearly, consider the gradual buildup of population immunity as vaccination gains prevalence. Instead of consistently pushing R to below 1, the increase in population immunity is offset by a relaxation of social distancing, keeping R around 1 and maintaining a significant rate of infection, a rate that will likely persist until vaccination prevalence approaches herd immunity levels [20]. Indeed, such a pattern has can be observed in countries such as the UK and the US (Fig 2).

These conditions create a potentially fertile breeding ground for vaccine escape [22]. Once a significant share of the population is vaccinated, a vaccine-resistant variant, which can potentially infect anyone, whether vaccinated or not, has a selective advantage relative to the wild-type strain, as the latter can only infect unvaccinated individuals. Since the wild type’s R is maintained around 1, this relative advantage translates into an absolute positive growth rate of R>1 for the resistant variant, allowing it–if it occurs by a random mutation–to quickly spread throughout the population. This, together with the large number of infections expected during the slow vaccination process, might create a high probability that a mutation will occur and take over the population. Such a mechanism for vaccine escape is, indeed, unique to situations in which mitigation involves both vaccination *and* social distancing, with the latter being relaxed in response to the progress of the former [19,20,22,23].

The straightforward solution is to avoid the extended period in which high vaccination prevalence coexists alongside a high rate of infection. Ideally, this would dictate a policy to vaccinate the entire population within a short period of time. Such a solution, however, ignores the main bottleneck to vaccine rollout, namely the inherent limitations on vaccination capacity. To overcome this obstacle, we propose a spatial vaccination strategy which will be shown to dramatically reduce the risk of vaccine escape, even under the existing constraints on the vaccination rate.

The proposed spatial strategy takes advantage of the geographic segregation that often characterizes the population distribution, and the fact that people mainly interact within the region they reside in. We propose to divide each country (or possibly a smaller geographic unit such as a state) into smaller regions that are sufficiently disconnected in terms of social interactions and then sequentially vaccinate one region at a time, thus concentrating the entire country’s vaccination capacity in order to quickly bring that region towards herd immunity. Such partitioning would replace the gradual accumulation of nationwide herd immunity. The obvious advantage is that the rapid achievement of herd immunity in each individual region should avoid the prolonged period of interaction between infected individuals and the vaccinated population. Thus, the dangerous combination of high infection rates (the source of mutations) and high vaccination rates (which provide an advantage to resistant strains) is dramatically reduced. Since the majority of infectious interactions are local in nature [24], namely they occur within a single region; cross-infection between regions is rare. Therefore, vaccinating all regions *one by one* may be able to facilitate a safe and rapid accumulation of local herd immunity in each region, until it is finally achieved for the entire population. The result will be to reach country-level immunity in roughly the same amount of time, but with a significantly lower risk of an escaping variant.

Other considerations may also be important in devising an effective vaccination strategy, and in particular the prioritization of the vulnerable population. We therefore also examine the application of spatial vaccination only after a uniform vaccination of up to 15% of the population (i.e. the most vulnerable groups). As we demonstrate, this has limited impact on the outcome of the proposed spatial vaccination strategy. The reason for this is that most of the additional risk of vaccine escape due to uniform vaccination occurs only once the vaccine coverage is well above 15%–prior to that the resistant variant’s selective advantage is small.

Spatial vaccination allows for additional (relatively low-cost) measures that further reduce the risk of vaccine escape and are not applicable or are too costly under a uniform vaccination regime. First, an effort can be made to identify and isolate infections by the resistant variant in the vaccinated areas. Such *variant contact tracing* is likely to be successful since in vaccinated areas, which are clear of wild-type infections, every short *infection chain* is highly likely to originate from the resistant variant. This measure is difficult to apply under the current vaccination regime, in which resistant variant infections may be hidden among the predominant wild-type infections. Second, the authorities can impose limitations on population movement between the vaccinated and unvaccinated regions. Such limitations would not be overly burdensome if the order of vaccination is wisely planned, with the goal of keeping the vaccinated and unvaccinated areas geographically contiguous, with one (moving) border between them. Third, the authorities may impose a short, *moving* lockdown that is applied in each region during or just prior to vaccination. Such a localized and brief lockdown can be more easily enforced relative to prolonged countrywide lockdowns, which impose a devastating individual and societal burden.

Finally, the spatial strategy is effective not only in mitigating vaccine escape, but also in reducing the overall number of infections. This is because, as vaccination progresses, the infections in regions that reach herd immunity will cease much earlier than under uniform vaccination. In fact, if the number of regions is sufficiently large, the total number of infections is reduced by close to 50%, since the infections in a region will on average end after half of the nationwide vaccination time.

Looking to the future, spatial vaccination may be useful if humanity will face a virus with two crucial properties. First, it is sufficiently harmful that–until vaccination can control it–social distancing must be imposed until a vaccine is developed. This is because it is the relaxation of social distancing following vaccination that generates the increased risk of resistant variants. Second, that R_0_ is not sufficiently high to prevent the vaccination from achieving herd immunity. The ability to quickly bring each specific region to herd immunity, so that infections cease there, is at the core of spatial vaccination. A future pandemic with these two properties may involve a completely new virus or a new variant of COVID-19 which escapes the immunity conferred by infection with the current strains or by vaccination, yet has a much lower R_0_ (Note than an escape variant can proliferate even if it is deficient relative to the current strain).

### Illustrative example

**Fig 3:**
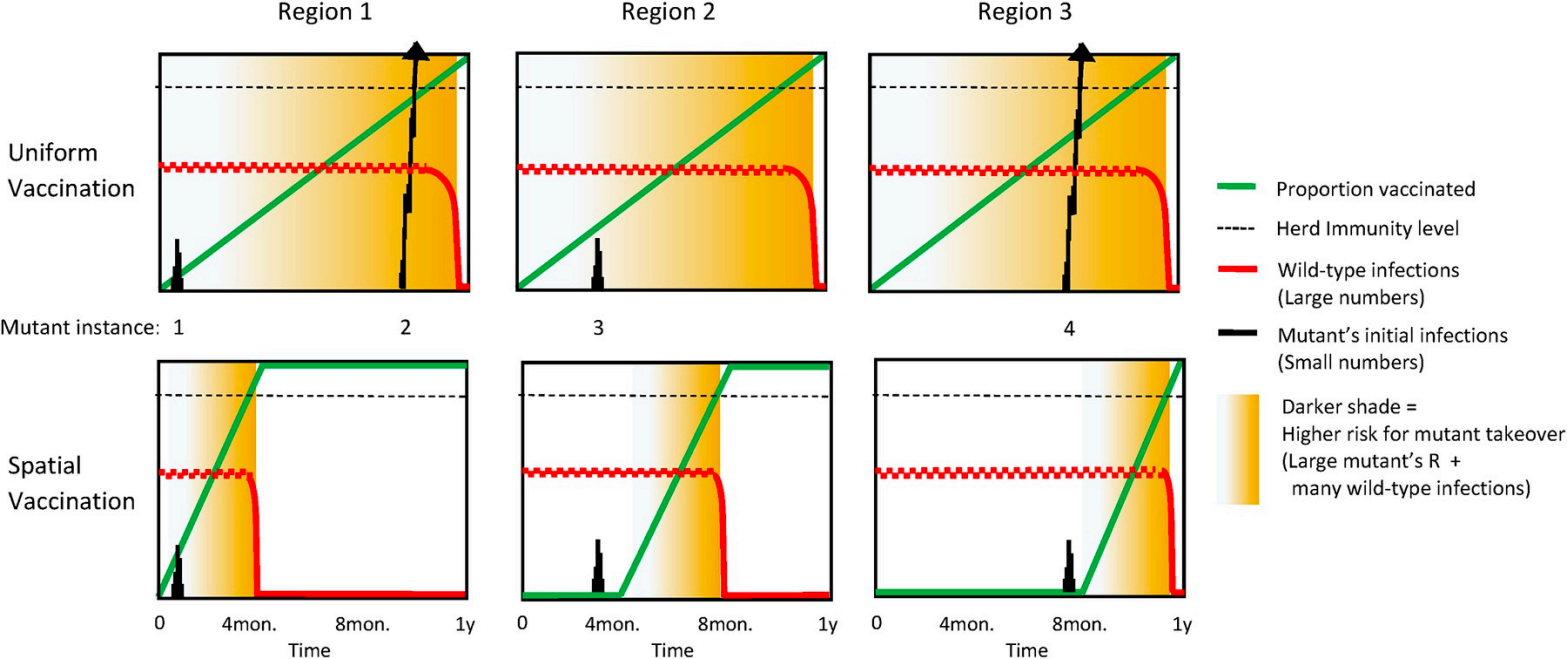
Illustration of uniform vaccination (top panels) vs. spatial vaccination (bottom panels) with 3 regions. As the proportion of the vaccinated population (green line) increases from 0 to 1, social distancing measures are gradually relaxed. Thus, the resistant variant’s R increases and with it the chance that a mutation will survive and become dominant (degree of orange shading). The large number of wild-type infections (red line) remains roughly constant until vaccination crosses the herd immunity level (dashed line). At that point, infections quickly decline and the risk of escape (orange shading) diminishes. Four potential instances of resistant mutants (1–4, black lines) are considered and the random walks of the (very small) number of infections in their early stages are illustrated. Under uniform vaccination (top panels), instances 2 and 4 occur in an environment with a large R (dark shading) and succeed in proliferating to numbers from which takeover is almost guaranteed. Under spatial vaccination (bottom panels), in instance 4 the mutation encounters a low R and dies out; mutation 2 does not even occur since in region 1 the stream of wild-type infections ends early on due to the rapid vaccination campaign. Instances 1 and 3 occur, under both scenarios, in environments with a small R and therefore die out.

To demonstrate the merits of the spatial vaccination strategy, in Fig 3 we illustrate the different vaccination scenarios for K = 3 regions over a one-year vaccination phase. First, we examine simultaneous vaccination, in which all three regions are treated concurrently (Fig 3A and 3C). The vaccine rollout (green line) occurs at the same pace in all three regions over the course of the year. During this period, as the population adapts its behavior to maintain R at around 1, we continue to observe a roughly constant stream of infection (red line) in all regions. This roughly stable level declines sharply once the herd immunity threshold is crossed (the dashed grey line). (Note that the different variables have different scales and are presented together in order to show the interplay between them.)

Sporadic instances of the resistant strain (the black lines) occur at random locations/points in time, denoted by 1,2,3 and 4. For example, instance 1 occurs in Region 1 one month after the start of the vaccination campaign (t = 1). At that time, the vaccine prevalence is still low. The resistant mutant thus has no significant selective advantage and therefore fails to proliferate. A similar pattern is observed in instance 3, which occurs at t = 3 months in Region 2. However, as the vaccination rollout progresses, the effective reproduction number of the resistant variant increases. Thus, the risk that a mutant will survive and proliferate, indicated by the shaded background, increases from low (light shading) to high (dark shading). This risk then rapidly drops again after herd immunity is surpassed and the infection streams cease. Indeed, instances 2 and 4, which occur at t = 9 months and t = 7 months, respectively, both have a significant selective advantage and are hence able to spread (steep ascent of black lines). These mutants eventually reach a number of infections beyond which it becomes inevitable that they will take over the population. As a result, we witness vaccine escape, and the simultaneous vaccination campaign fails.

We next consider the same scenario, except that instead of vaccinating the entire population over the course of one year, we sequentially vaccinate the regions in three rounds, with each campaign lasting four months (Fig 2D–2F). The two escaping variants in instances 2 and 4 are now averted. Instance 2 does not occur since at t = 9 months Region 1 is already cleared of the virus. Instance 4, on the other hand, also occurs in this scenario. However, it fails to proliferate since at the time of its appearance, i.e. at t = 7 months, Region 4 has not yet begun to vaccinate, and therefore, in contrast to the previous scenario, this mutant has a low reproduction number (light rather than dark shading), and poses little risk of escape.

Hence, by splitting the yearly nationwide vaccination cycle into shorter regional ones, we significantly reduce the risk of vaccine escape, thus replacing the extended high-risk time window of potential vaccine escape (Fig 2A–2C) with a sequence of narrow time windows, one in each region (Fig 2D–2F).

## 2. Results and discussion

We consider the susceptible-infected-recovered (SIR) model and augment it with vaccination and mutations (see Methods section) [25,26]. The population is assumed to adapt its behavior by means of social distancing in order to maintain R at approximately 1. As a result, we observe a roughly constant rate of new infections, which we set at 10% of the population per year. Following one year of such dynamics, we introduce the vaccine whose rollout and production rates make it possible to vaccinate the population within one additional year (For simplicity, we consider one-dose vaccination and immediate full immunity following vaccination). The vaccinated territory (a country, state or other distinct geographic unit) is divided into K regions such that, on average, only C = 1% of a person’s interactions are out-of-region. We vaccinate all regions sequentially up to 80% coverage. Hence, a specific region k (k = 1,…,K) can be in one of three states at any given moment: pending vaccination as it awaits its turn, undergoing vaccination, and post-vaccination, at which point the campaign progresses to vaccinating region k+1. Regions that are pending vaccination continue to accumulate infections at the constant rate of 10%/year. Similarly, regions undergoing vaccination also experience a constant 10% annual infection rate until they reach herd immunity. This is a consequence of their adaptive social distancing, which is relaxed as immunity accumulates [19,20]. After herd immunity is surpassed, infections quickly decrease and social distancing ceases.

Mutations occur with a small probability, denoted by *μ*, at each infection event. Hence, an individual carrying the wild type may infect a susceptible individual who may then, with probability *μ*, acquire a vaccine-resistant strain. (This simplistic model of resistant variant emergence ignores various biological facts, which are discussed in detail in Section 2G.) If such a mutant occurs, we model the process of subsequent infection using a discrete random walk process until it either dies out or takes over (see below). For expositional simplicity, we assume for now that the resistant strain (1) has the same basic reproduction number as the wild-type strain, (2) is fully resistant to the vaccine, and (3) cannot infect those who have recovered from prior infection with the wild type. These assumptions are relaxed in Section 2F.

### A. The basic simulation

We now turn to computing the probability of vaccine escape. The risk of such an event depends, first and foremost, on the probability of a resistant mutation *μ*. It is worth emphasizing that vaccine escape is a global problem, since a mutation in any country, even outside the territory currently being vaccinated, will eventually reach all countries and potentially undermine the vaccination campaign globally. Therefore, even if *μ* is extremely small, given the large-scale transmission of infection worldwide, the risk is not negligible. To account for this in our analysis, we focus not only on instances of mutation within the population of our simulated territory, but rather on *all* potential mutations on a global scale, i.e. among a population of *N* = 7.8×10^9^. The challenge is that the value of *μ* is unknown. Thus, while the probability of a single point mutation in SARS-COV-2 can be computed from its genetic properties, the probability that a *combination* of mutations will generate a variant resistant to the current vaccines cannot be calculated. We thus consider a broad range of mutation probabilities, spanning three orders of magnitude, from extremely rare (*μ* = 10^−10^) to highly frequent (*μ* = 10^−7^). Under a mutation rate of *μ*<10^−10^(<*N*^−1^), it is unlikely that even one mutation will occur, even in the case that 10% of the world population is infected over the course of one year, and therefore escape is very unlikely. With a rate of *μ*>10^−7^ mutants are present at any given moment among the estimated 10^7^ infected individuals in each infection cycle, and therefore an escape is almost certain even under instantaneous vaccination. For each value of *μ* within the range of interest, namely 10^−10^<*μ*<10^−7^, we simulated 50000 realizations and calculated the escape probability P from the number of realizations in which a resistant strain was able to propagate and take over the population. (Additional parameter values for the simulation are: R_0_ = 4; infection cycle of 4 days.)

**Fig 4:**
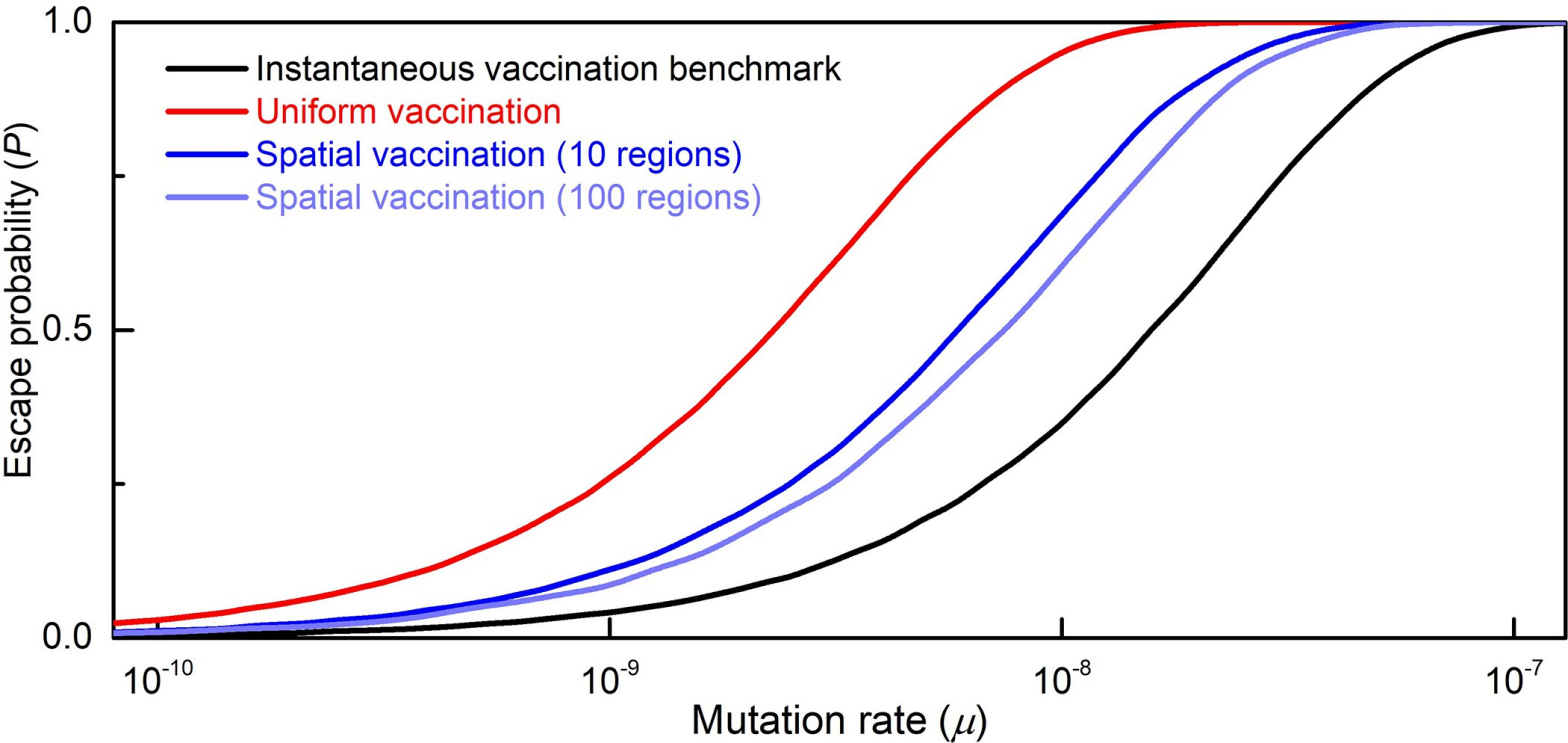
Probability of vaccine escape as a function of the mutation rate under various vaccination regimes. For mutation rates between 10^−10^ and 10^−7^, the probability of escape under the current regime of uniform one-year vaccination (red curve) is far higher than under the benchmark of instantaneous world vaccination (black curve). Spatial vaccination with K = 10 regions (dark blue curve) subject to the same one-year constraint restores about 50% of the excess risk. Increasing the number of regions to K = 100 (light blue curve) generates a modest additional gain.

The results are presented in Fig 4. We first consider the ideal scenario in which the entire population is vaccinated instantaneously, i.e. in a single day (the black curve). Under these conditions, the only risk of vaccine escape originates from mutations that occurred prior to the vaccination campaign. We find that for *μ*>10^−7^ escape is practically unavoidable (*P* = 1). This marks the upper bound on *μ*, beyond which we cannot hope to avert vaccine escape. Of course, instantaneous vaccination is unattainable in practice. Therefore, the black curve in Fig 4 represents our ideal benchmark to which we will compare the different strategies.

We next consider simultaneous vaccination over the course of one year (the red curve). As expected, we find that the extended vaccination period exhibits a higher probability of escape. Specifically, we observe a window of 10^−7^>*μ*>10^−10^ in which the year-long vaccination campaign leads to significant additional risk.

Finally, we consider our proposed spatial vaccination strategy, which divides the vaccinated territory into K = 10 regions, each with ~10% of the population (the dark blue curve). The results clearly show that spatial vaccination eliminates a large part of the excess risk, bringing us closer to the desired benchmark. For K = 100 (light blue curve), we only observe a marginal additional benefit, which is an indication of the bounds on the potential benefit of spatial vaccination.

#### A measure for differences in escape risk

Obtaining a single number that measures the differences in escape risk under the various vaccination regimes is not straightforward. For example, the difference in escape probability is large for intermediate mutation rates but small for very low or very high ones. Using the ratio of escape probabilities does not solve the problem either since again its value is not constant, becoming smaller at high mutation rates. We therefore propose a more suitable metric for comparing vaccination regimes: the *difference in the respective mutation rates that lead to the same escape probability under the two regimes*. Below, we formally prove that this measure is *constant* across different escape probabilities. Thus, the curves in Fig 4 are lateral shifts of one another, and one number, i.e. the horizontal distance, suffices to compare two regimes.

Formally, given a configuration *c* (vaccination regime and model parameters except for *μ*), denote the stream of new infections at time *t* by *x*_*k*_(*t*, *c*) and the probability of takeover by a mutant that occurs at time *t* (and assuming that no other mutant occurs any time) by *p*_*k*_(*t*, *c*). The probability of escape (i.e. at least one mutant occurring and taking over) is: $P(c,\mu )=1-\prod_{t=0}^{T}{\left(1-p_{k}(t,c)\right)}^{{\mu x}_{k}(t,c)dt}$, where ∏ here denotes the geometric integral—the continuous version of the usual product sign.

**Claim:** If *P*(*c*_1_, *μ*_1_) = *P*(*c*_2_, *μ*_2_), then *P*(*c*_1_, *αμ*_1_) = *P*(*c*_2_, *αμ*_2_)

**Proof:** Taking the natural logarithm of 1−*P*(*c*, *μ*) computed above we obtain:

$$ln(1-P(c,\mu ))=\mu \int_{t=0}^{T}x_{k}(t,c)ln(1-p_{k}(t,c))dt.$$

Thus, *P*(*c*_1_, *μ*_1_) = *P*(*c*_2_, *μ*_2_) is equivalent to:

$$\mu_{1}\int_{t=0}^{T}x_{k}(t,c_{1})ln(1-p_{k}(t,c_{1}))dt=\mu_{2}\int_{t=0}^{T}x_{k}(t,c_{2})ln(1-p_{k}(t,c_{2}))dt$$

while *P*(*c*_1_, *αμ*_1_) = *P*(*c*_2_, *αμ*_2_) is equivalent to:

$${\alpha \mu }_{1}\int_{t=0}^{T}x_{k}(t,c_{1})ln(1-p_{k}(t,c_{1}))dt={\alpha \mu }_{2}\int_{t=0}^{T}x_{k}(t,c_{2})ln(1-p_{k}(t,c_{2}))dt.$$

Clearly, the first equation implies the second. QED.

#### Quantifying the differences in escape risk

With this metric in hand, we can quantify the results of the simulation above. The excess risk due to a uniform one-year vaccination regime relative to the instantaneous vaccination benchmark is 0.8 orders of magnitudes. That is, uniform vaccination increases the escape risk to the same extent as in the case that mutations were 10^0.8^ = 6.3 times more frequent. Spatial vaccination with K = 10 regions restores 0.42 orders of magnitudes or in other words about 50% of the excess risk. Thus, moving from uniform to spatial vaccination allows for a mutation rate that is 10^0.42^ = 2.6 times higher for the same escape risk.

The importance of reducing the risk by 0.4 orders of magnitudes depends on our assumption regarding the probability distribution of *μ*. We discuss ways to address this issue in Section 2G. Note also that this result hinges on the simplistic assumption that resistant variants are as infectious as the wild type, which was made for purposes of simplifying the exposition. In Section 2F, we make a more realistic assumption that the escape variant has a lower basic reproductive number than the wild type in view of the fact that immune evasion may require deleterious mutation in the virus [27,28]. Under such assumptions, we obtain an even stronger advantage of spatial over uniform vaccination. Moreover, we show in Section 2C that using spatial vaccination makes it possible to employ a number of complementary measures that are infeasible under uniform vaccination. These measures improve the effectiveness of the spatial strategy to an even greater extent.

#### Reducing the total number of infections

Apart from reducing the risk of vaccine escape, spatial vaccination has a second advantage: a dramatic reduction in the number of infections with the wild-type strain. This is because in vaccinated regions which reach herd immunity, the stream of infections ceases much earlier than under uniform vaccination. As a rough approximation, if the number of regions is sufficiently large, the total number of infections is reduced by close to 50% as compared to the uniform vaccination regime. To see this, note that region k = 1…K experiences infection from time 0 until time k/K and thus, for the average region infections end at time (1+K)/K, which approaches ½ for large K. Thus, for the average region, infections last for half a year rather than one year, as under uniform vaccination.

### B. The partition into regions

The setting of the parameter K, i.e. the level of spatial partitioning, involves a critical tradeoff. On the one hand, the larger K is, the smaller each region will be and therefore the faster will be a region’s attainment of herd immunity. This limits the time window in which vaccinated and infected individuals are interacting and thus reduces the escape risk for each region. At first glance, it would thus seem preferable to partition the territory into as many regions as possible. However, increasing K comes at a price: if the regions are too small they may not be socially separable. For example, if we try to partition a city or a densely populated country, individuals from different regions are likely to interact, undermining the potential benefit of the spatial strategy. We therefore need to seek an optimal balance between the number of regions (K) and the level of inter-regional coupling (C).

**Fig 5:**
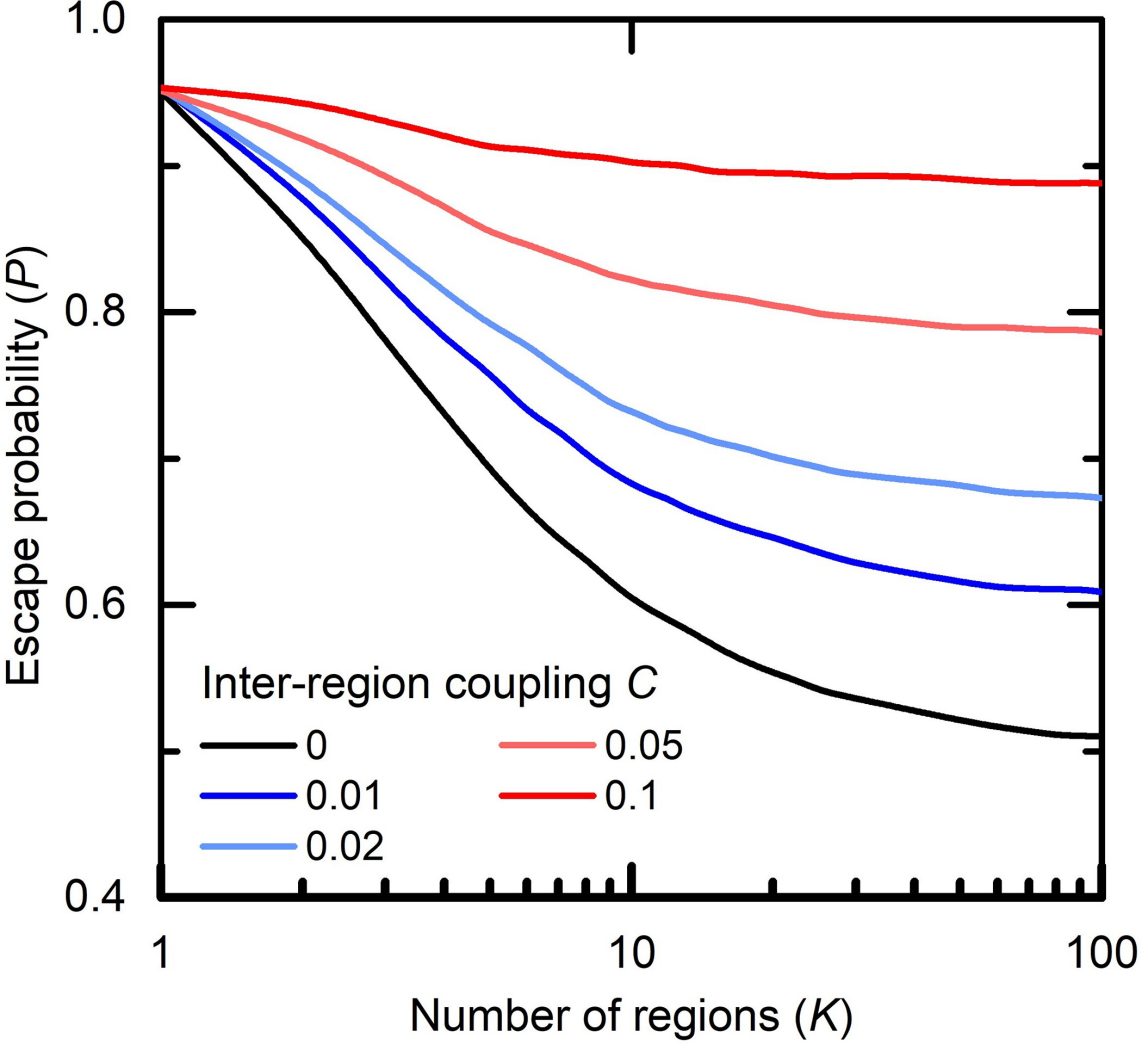
**The number of regions vs. the separation between regions** (for *μ* = 10^−8^): In the case of a contact ratio of 1% (blue line) or 2% (light blue line) we observe a steep reduction in the probability of escape as the number of regions K increases up to about 20; the benefit of increasing K further is limited (the curves almost flatten out). With a contact ratio of 5% (orange) or 10% (red) the benefit of increasing K is small, so that C = 5% and K = 10 is equivalent to C = 1% and K = 3. The black line indicates the ideal limit at which there are no contacts between regions (C = 0).

To systematically examine this tradeoff, in Fig 5 we present the escape probability P vs. the number of regions K, under various values of the coupling parameter C. We first observe that the benefit from increasing K reaches saturation for K > 20, which is in line with our previous results in Fig 4, which showed only a marginal gain from increasing K from 10 to 100. As expected, we also find that as C increases the effectiveness of the spatial strategy declines. Specifically, for C > 5% the spatial vaccination strategy ceases to offer a significant benefit.

This makes it possible to establish guidelines for spatial partitioning. Each country or equivalent geographic unit should seek to establish naturally separated regions that satisfy *C*≅1%, namely that only 1% of the population’s interactions are out-of-region. Therefore, partitioning for example a city or large metropolitan area is likely to be inefficient, while separating at the county, province or state level is more likely to achieve the desired effect.

It is important to note that even if a considerable percentage of the population lives in a large metropolitan area that cannot be divided for purposes of partial vaccination, it is still beneficial to divide the remaining population according to region. This is because the escape risk from each region is cumulative. That is, the factor that determines the probability of a resistant variant takeover is the proportion of infections (i.e. mutation opportunities) that will occur while there is a highly vaccinated population in that region (resulting in a high reproduction rate for resistant variants). Thus, for example, if 25% of the population lives in a metropolitan area that takes three months to vaccinate, and the remaining 75% can be divided into 9 regions that take one month each to vaccinate, then 75% of the K = 1 probability is reduced by the factor that K = 12 generates, and 25% is reduced by the factor that K = 4 generates.

#### Estimating C

To estimate the value of the inter-regional coupling parameter C, we rely on recent observations which show that–almost universally–mobility fluxes decay with distance according to *d*^−2^, an inverse square law [24]. We can use this to evaluate the desired radius *ρ* of the vaccination regions. Specifically, we seek the percentage *C* of individuals that, within the typical duration *τ* of an infection cycle, travel a distance that exceeds *ρ*, and hence can potentially cross-infect between two or more regions. Denoting the average individual trip within the *τ*-timeframe by $\bar{\rho }$, we can use the inverse square law to approximate $C(\rho )∼{\left(\frac{\bar{\rho }}{\rho }\right)}^{2}$, thus capturing the fraction of the population that travel beyond a radius *ρ* within the transmission window *τ*. Extracting *ρ* from this relationship we arrive at $\rho =(1/\sqrt{C})\;\bar{\rho }$. Therefore, to achieve, for example, *C* = 5%, we must set $\rho \approx 5\bar{\rho }$, five times the typical individual distance travelled. For *C* = 1%, we get $\rho \approx 10\bar{\rho }$, a ten-fold factor. Taking $\bar{\rho }∼30$ km [29], we obtain *ρ*~10^2^ km in order to ensure *C* within the range of 1−5%. For a typically-sized country, this is well within the desired boundaries of *K*~10 regions, which captures the safe operating zone shown in Fig 5.

Note that while the above analysis considers the coupling parameter *C* under unrestricted mobility patterns, in practice, we can employ active interventions to drive *C* towards a desired value (see the discussion in Section 2C).

#### The role of inter-region coupling and the order of vaccination

To better understand the role of inter-region coupling C, consider a region that has already been vaccinated. Having reached local herd immunity, infections in that region have ceased and hence, given adaptive social behavior, practically all restrictions have been lifted. Due to the vaccination, the wild-type’s reproduction number is kept below one, and thus any wild-type infection that arrives from another region dies out quickly. However, if the resistant strain arrives, then, absent social distancing, it benefits from a very high R (which equals R_0_) and therefore quickly ignites a new breakout of infection. Note that for any region, this risk only exists post-vaccination since prior to vaccination social distancing restrictions limit the ability of a mutant to take off, and incoming infections are negligible relative to the flow of within-region infections.

The implication is that interactions between vaccinated regions or between unvaccinated regions do not pose a problem. What matters is the separation, at each point in time, between the vaccinated regions and the not-yet-vaccinated regions. Thus, the partitioning into regions and the order of vaccination should be designed such that bordering regions, which are likely to have a high level of contact, are vaccinated adjacently in time, so that there is one “moving” border between the vaccinated and unvaccinated regions. Therefore, C should be interpreted as the ratio of an average person’s interactions with people on the other side of the “border” to his interactions with people on his own side of the border. Under this interpretation, C∼1% or lower appears to be reasonable.

The spatial vaccination strategy makes it possible to employ a few simple and relatively low-cost complementary measures that reduce the risk of vaccine escape even further, including travel limitations between vaccinated and unvaccinated regions, contact tracing for resistant variants and temporary regional lockdowns during vaccination. Importantly, these measures are not applicable or are too costly under a uniform vaccination regime. We assess the effectiveness of each of these measures by repeating the simulation carried out in Section 2A with the necessary modifications (see formal treatment in the Methods section).

#### Limitations on travel between vaccinated and unvaccinated regions

The authorities can impose temporary limitations on travel between vaccinated and not-as-yet vaccinated regions. This will avoid the potential spillover of mutations from unvaccinated to vaccinated regions. In the latter restrictions are lifted and any entering mutant will enjoy a large reproduction number and a high likelihood of success. Indeed, the coupling between same-type regions has only little effect and thus the effect of reducing travel just between vaccinated and not-as-yet vaccinated regions is almost the same as that of a reducing travel between all the regions (i.e., reducing the coupling parameter C). Importantly, if the vaccinated regions as a whole and the unvaccinated regions as a whole are each kept contiguous, with one moving border between them as explained above, then the limitations on travel–only across that border–will not be overly burdensome.

**Fig 6:**
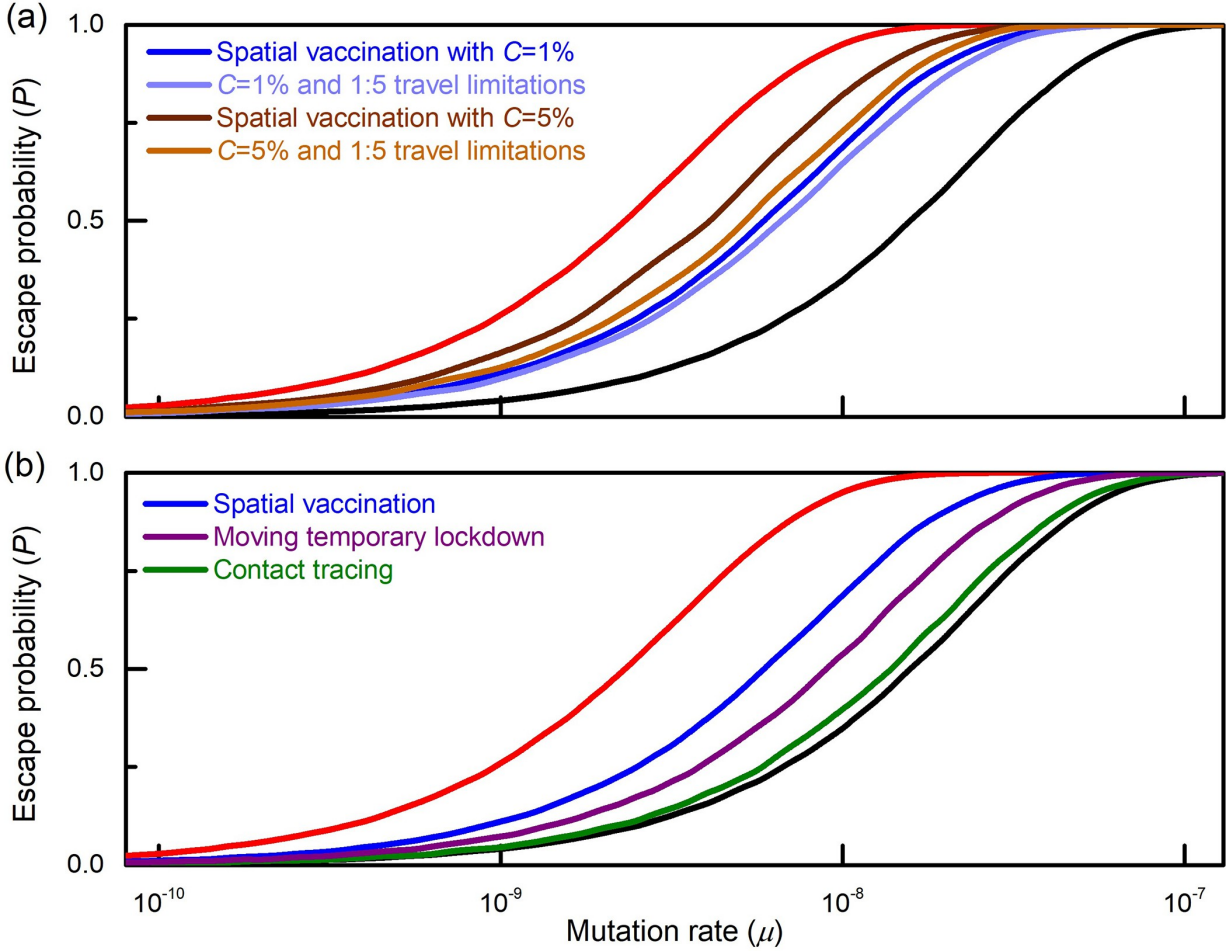
Complementary measures. **(a) Restricting travel** between vaccinated and unvaccinated regions by a factor of 5 reduces the probability of escape (light brown vs. dark brown and light blue vs. blue curves). In particular, if the initial inter-region coupling is large (C = 5%, dark brown), the outcome under travel restrictions (light brown) is almost as good as in the case of C = 1% (blue), i.e. implying that travel between same-type regions has little effect. **(b)** A short **temporary lockdown** of the region being currently vaccinated, that reduces the effective reproduction number there to 0.8 (rather than 1) reduces the escape probability (purple vs. blue curves). **Contact tracing** for the resistant variant–which is assumed to be feasible only in vaccinated regions that are almost clear from wild-type infections, and in that case reduces the variant’s effective reproduction number R by a factor of 2 –has a dramatic effect on the escape probability (green vs. blue curves).

Fig 6A presents the effect of travel limitations between vaccinated and unvaccinated regions. The red and black lines represent, as they did in Fig 4 above, the escape probabilities under uniform vaccination and instantaneous vaccination, respectively. We observe that the probability of escape under spatial vaccination with K = 10 regions and c = 1% (blue curved) is reduced when travel is restricted by a factor of 5 (light blue curve). We also consider the case of C = 5% (brown curve). We see that restricting travel between vaccinated and unvaccinated regions by a factor of 5 (light brown) is almost as effective as having C = 1% initially (blue curve).

#### Contact tracing of the resistant variant

Recall that, in vaccinated regions, the wild-type strain has been eradicated and moreover, any wild-type infection imported from other regions dies quickly due to the high vaccination level. Therefore, any infection chain among vaccinated individuals–even if short–is highly suspected of belonging to the resistant strain. As a result, contact tracing targeted specifically at resistant variants will be highly effective. It is worth emphasizing that variant contact tracing can be effective *only* under the spatial vaccination strategy. Indeed, under uniform vaccination resistant-variant infections are hidden among the many wild-type infections, and hence contact tracing becomes infeasible.

We repeat the simulation under the assumption that contact tracing of infections with the resistant strain can be effectively applied only when–due to vaccination–the stream of new wild-type infections in region k falls to below 1/10 of the pre-vaccination level and that when contact tracing is implemented it reduces the resistant variant’s reproduction number by a factor of 2. The outcome is depicted by the green line in Fig 6B.

#### A moving temporary lockdown

A third measure that can further reduce the escape risk under spatial vaccination is the application of a short-term, moving lockdown of the region currently being vaccinated. Indeed, a month-long lockdown will be more acceptable to the population than the year-long lockdown required to achieve the same objective under uniform vaccination, and it has a much better cost-benefit ratio. Fig 6B (purple line) presents the outcome under stricter temporary restrictions, only in the region being vaccinated, such that the reproduction number there is further reduced to 0.8 (rather than 1).

Note that an alternative scenario which leads to the same outcome is a delay in the behavioral response to the increased level of vaccination. Since the increasing beneficial effect of vaccination is counteracted by a delayed relaxation of social distancing, the region being vaccinated will enjoy a reproduction number of less than 1.

### D. Practical and ethical concerns

Although a spatial vaccination strategy will reduce the likelihood of escape variants, policy makers may have other considerations as well. Thus, they may also care about the total level of infection among the population, including of course infection with the current variant. Therefore, they may want to vaccinate certain populations sooner, either due to their increased vulnerability or higher contact rate. Furthermore, they may want to adopt a policy that is fair and also perceived to be so.

#### The total number of infections

It turns out that the spatial strategy is effective not only in mitigating vaccine escape, but also in reducing the overall number of infections. This is because, as vaccination progresses, the spread of infection in regions that reach herd immunity will cease much earlier than under uniform vaccination. In fact, if the number of regions is sufficiently large, then the total number of infections will be reduced by close to 50%, since infections in a specific region will on average end after half of the nationwide vaccination time.

On the other hand, spatial vaccination might slow the vaccination process. Thus, there are two kinds of limitations on the ability to concentrate effort in small regions in order to accelerate vaccination: vaccine production capacity and logistic capabilities. The former is in fact a global constraint and therefore there is no difficulty in directing global output to specific regions. Therefore, this constraint does not limit the effectiveness of the spatial strategy. The second and binding constraint is dependent on logistics, such as how easily medical personnel can be moved from one region to another, or the availability of facilities in which vaccination can take place. Thus, the overall pace of vaccination might be slowed by adopting the spatial strategy, and policymakers need to take this into account. However, the experience with COVID-19 shows that although vaccination is slow at first, the pace quickly improves. Therefore, it may turn out to be more effective to create the necessary logistics infrastructure and move it from region to region, thereby saving time.

#### Prioritizing selected populations

Considerations of morbidity and mortality might argue for prioritizing the vaccination of vulnerable populations, such as the elderly or those with certain pre-existing conditions, while the desire to lower infection rates may lead to earlier vaccination of populations with high contact rates, such as doctors or teachers (see [3]). We therefore consider a mixed vaccination regime, in which a policy of uniform vaccination is initially adopted for some proportion of the population (the prioritized group), followed by spatial vaccination of the remaining population.

**Fig 7:**
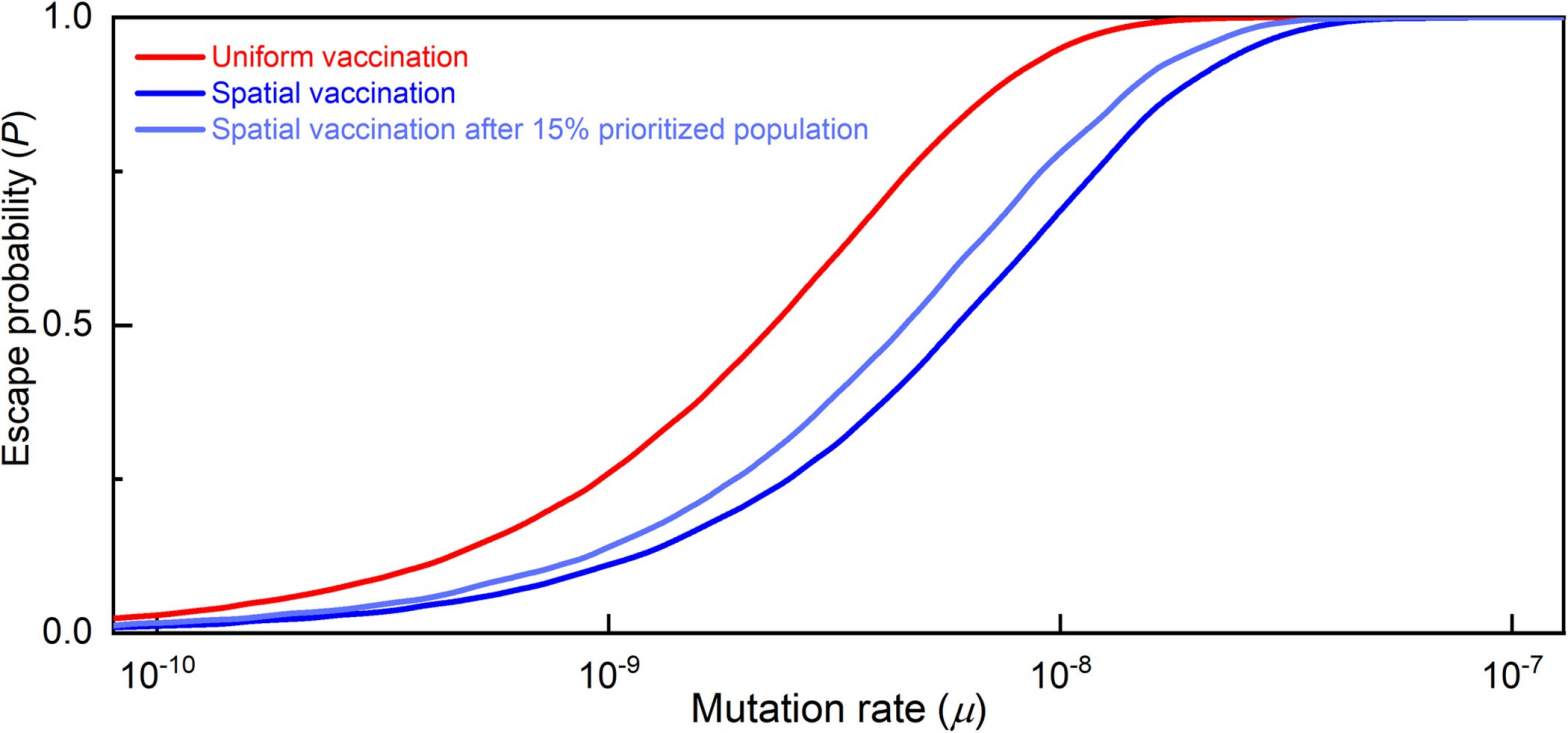
Prioritizing a defined population. Allowing 15% of the population (such as the more vulnerable) to be vaccinated first and only then proceeding to the spatial vaccination strategy eliminates only a small part of the benefit from spatial vaccination.

The outcome is presented in Fig 7. It can be seen that in the case where the prioritized group accounts for 15% of the population, most of the benefit of spatial vaccination is preserved. This is because most of the additional risk of vaccine escape due to uniform vaccination occurs only once the vaccine coverage is well above 15%; prior to that the resistant variant has little selective advantage, if any.

#### Fairness considerations

Is the spatial vaccination policy “fair”? Section 2B described the benefit of a spatial strategy in which a minimal moving border is maintained between vaccinated and unvaccinated regions. However, people may prefer a policy that randomizes equally across the population, or within groups that are ordered according to some clear policy consideration. Note that the minimal-border policy does not preclude randomizing since the campaign can proceed in any direction–for example, from north to south or south to north–thereby maintaining some degree of fairness. However, this is not the case for a policy that prioritizes groups by, for example, degree of vulnerability, and hence the spatial vaccination policy may not be suitable when the ethical considerations of “fairness” dominate.

### E. Viral environments in which spatial vaccination is advantageous

#### Range of R_0_

The advantage of spatial vaccination crucially depends on the feasibility of bringing regions to herd immunity, thus putting an end to infection and to the potential emergence of mutants. In the ideal case of a vaccine that provides perfect immunity against the wild type, achieving herd immunity requires that a proportion of at least 1-1/R_0_ of the population be vaccinated or infected. That level might be hard to reach in the presence of vaccine hesitancy or of populations that cannot be vaccinated.

Thus, in the case of the COVID-19 pandemic, the spatial vaccination strategy would have been advantageous in the case of the original Wuhan strain (R_0_ of between 2 and 3) and the Alpha variant (R_0_ of between 3 and 4). Since in the case of these strains the Moderna and Pfizer vaccines were almost full proof in preventing transmission, herd immunity could have been reached with between 60% (Wuhan) and 70% (Alpha) of the population being vaccinated. However, in the case of variants such as Delta (R_0_ of between 5 and 8) or Omicron (R_0_ well above 10 and significantly reduced vaccine effectiveness) herd immunity is not feasible before a large proportion of the population is infected, and thus spatial vaccination is not helpful.

**Fig 8:**
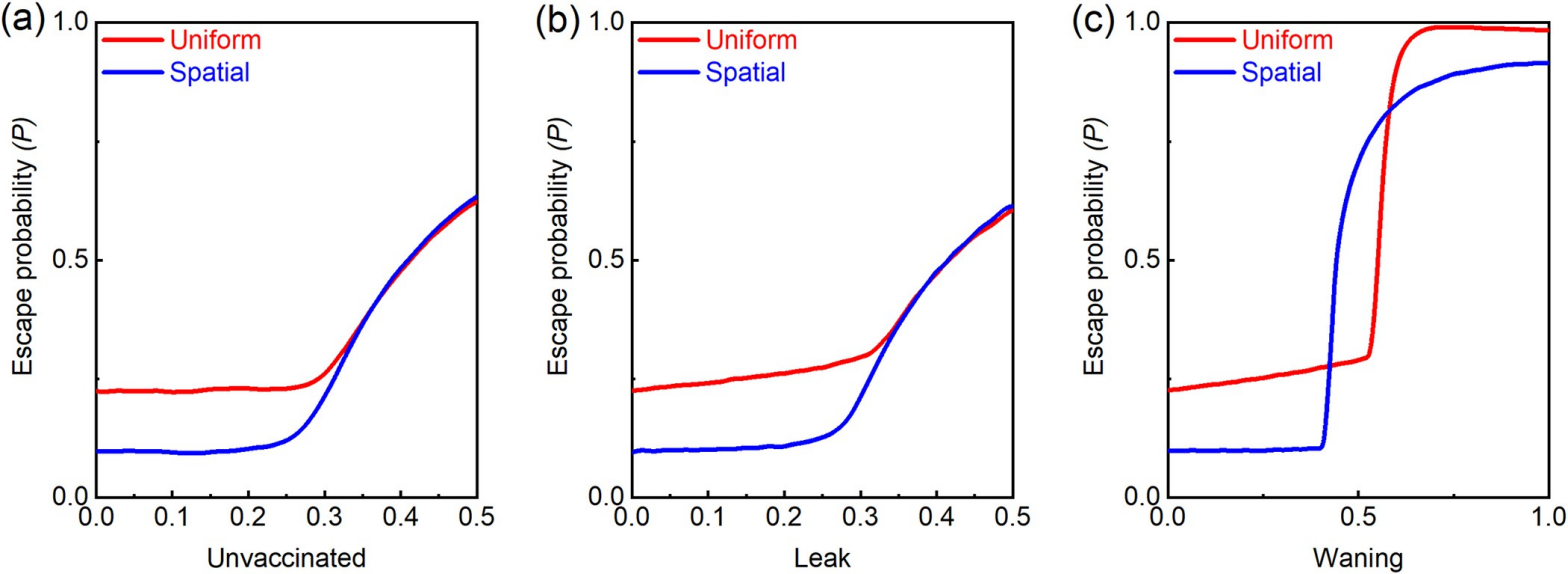
The effect of incomplete or imperfect vaccination (for *μ* = 10^−9^). **(a)** When the proportion of unvaccinated people exceeds 1/R_0_, such that herd immunity is not attained, the advantage of spatial vaccination is lost. **(b)** A similar outcome is achieved if the vaccine is not fully effective in preventing transmission (“leakage”). **(c)** If the vaccine’s effectiveness wanes over time, the advantage of spatial vaccination is lost for an intermediate rate of waning.

The effect of the unvaccinated proportion of the population on the probability of vaccine escape (in the case of a vaccine that provides perfect immunity against the wild type and with R_0_ = 4) is described in Fig 8A. It can be seen that the benefit of spatial vaccination vs. uniform vaccination diminishes once the proportion of the unvaccinated exceeds 0.25 (which is about 1/R_0_) and is completely lost above 0.35.

The difficulty of reaching herd immunity will be exacerbated when the vaccine is imperfect. This can occur in two cases: when the vaccine is “leaky,” i.e., less than fully effective in preventing transmission, or when its effectiveness wanes over time.

#### “Leaky” vaccines

Even at a reasonable level of R_0_, herd immunity is harder to reach with “leaky” vaccines. One would expect that with vaccines that have greater than 95% effectiveness, as in the case of the Moderna and Pfizer vaccines, the advantage of the spatial vaccination strategy will remain significant, though not in the case of less effective vaccines. Fig 8B describes an extension of our model to a leaky vaccine (assuming no unvaccinated). The qualitative effect of employing an imperfect vaccine is similar to that of having less than 100% vaccinated (Fig 8A).

#### Vaccine waning

A related form of vaccine imperfection is that its effect may wane over time. Thus, vaccine effectiveness will be diminished for those vaccinated early by the time the entire population has been vaccinated. Since our focus is whether herd immunity can be achieved, the question becomes the extent to which the vaccine’s effectiveness wanes during the time it takes to vaccinate the entire population.

The effect of waning is demonstrated in Fig 8C. The waning parameter (on the horizontal axis) captures the speed of decline (assumed to be linear) in vaccine effectiveness (leakage) over a period of one year. The simulation assumes that it takes one year to vaccinate the entire population and that it is re-vaccinated yearly. Thus, while the group of people vaccinated at time *T* is fully protected at that point in time, the average vaccine effectiveness for them at time *T*+*t* (where 0<*t*<1 is the time in years after *T*) is 1−*wt* due to waning. At time *T*+1 the group is vaccinated again and vaccine effectiveness returns to 1, and so forth. We immediately see that as long as the waning is not excessively fast (not more than about 35% per year), spatial vaccination dominates uniform vaccination, as in the case without waning.

At some level of waning, the ability to reach herd immunity is lost. With uniform vaccination, this occurs when *w* reaches ½. To see this, note that after one year or more, at each point in time the population is always a mix of people who were vaccinated at different dates, from one year earlier (so that their leakage is *w*) to just now (so that their leakage is 0). On average, the leak is *w*/2, and if it is greater than 0.25 (= 1−*R*_0_) then herd immunity is not achieved. Thus, at *w*>½ we see a jump in the escape probability.

In the case of spatial vaccination, the loss of herd immunity occurs earlier. This is because the groups (which are differentiated by the period of time since their vaccination) do not mix, unlike in the case of uniform vaccination. Thus, the average protection in the regions vaccinated early on is below herd immunity, even when *w*<½. Importantly, note that when a region falls below the herd immunity level, it is quickly re-infected through travel to and from other regions where infection is occurring. Unlike the small chance that a rare mutant will move between regions, it is very likely that some people infected with the wild type will travel across regions, unless there are severe limitations on movement.

Thus, there is an intermediate range of *w* where uniform vaccination dominates spatial vaccination. However, once *w* is sufficiently high that herd immunity is lost even under uniform vaccination, the outcome under spatial vaccination dominates. The reason for this is straightforward: under uniform vaccination all regions have the same average protection, which is a little below herd immunity, implying both a maximal flux of infections and a high R_0_ for the escape variant. Under spatial vaccination, some regions have higher than average protection while others have less than average. Thus, some regions will be above the herd immunity level (and therefore will have no infections) while regions with lower protection will have the same number of infections but with a lower R_0_, which translates into a lower likelihood for a new escape variant to survive. Both effects are in favor of spatial vaccination.

To compete the picture, it is worth emphasizing that if waning is fast and the disease is sufficiently severe that society is willing to bear the costs of achieving extinction, then spatial vaccination–coupled with a severe travel ban between vaccinated and unvaccinated regions–becomes the preferred policy. By quickly vaccinating regions successively, herd immunity is achieved before waning precludes it. And when protection in those regions declines to below herd immunity because of waning, reinfection is prevented thanks to the restrictions on movement between regions.

### F. Effect of parameters

To simplify the exposition, we have so far assumed that an “ideal” escape variant, which (1) has the same basic reproduction number as the wild type, (2) cannot infect people with antibodies from a prior wild-type infection (i.e. we assumed two-way cross immunity) and (3) is fully resistant to the vaccine. In reality, there are a large number of conceivable combinations of mutations, in which each of the above assumptions may be violated. In this section, we examine the consequences of relaxing these assumptions.

#### Escape is costly

It is reasonable to assume that a vaccine-resistant variant will be deficient relative to the wild type with respect to its ability to infect unvaccinated individuals. This follows from our definition of the wild type, as the dominant variant prior to vaccination, i.e. the one with the maximal R_0_ vs. unvaccinated among all variants. The resistant variant, which is selected from a strict subset of the variants–those resistant to the vaccine, will typically have a lower R_0_ vs. unvaccinated. Moreover, vaccines such as Moderna’s and Pfizer’s SARS Cov2 vaccines are designed to target a conserved protein, such as the spike protein. This design is based on the rationale that targeting conserved and highly essential viral proteins is likely to impose a high cost on immune-evading mutations [30]. (Mutant binding data of the type produced in [13,31] may be used to assess the existence and sign of potential correlation between, for example, antibody and receptor binding.)

**Fig 9:**
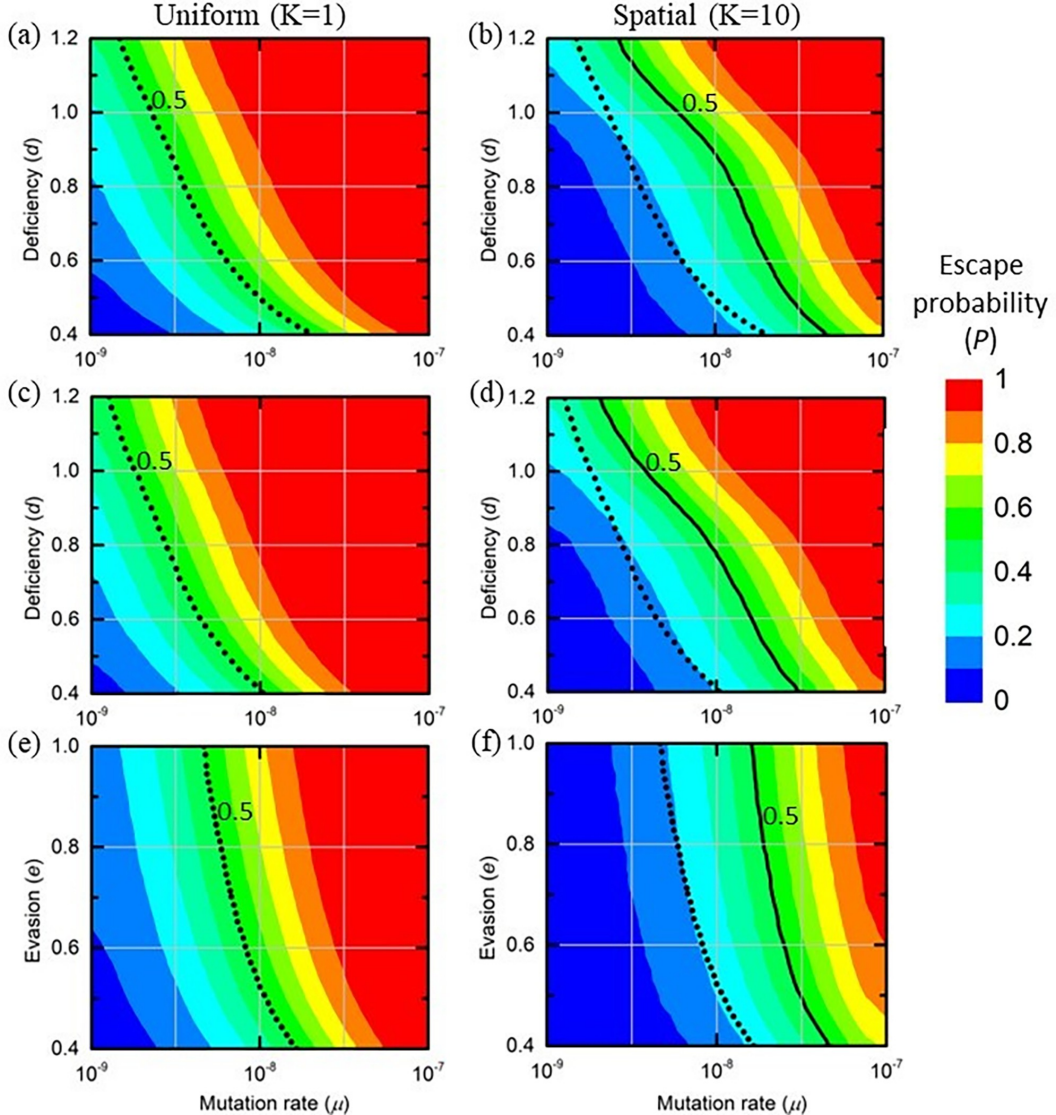
Probability of escape for different mutation rates under spatial uniform vaccination (left) vs. spatial vaccination (right). (a,b) As a function of the variant’s deficiency ratio *d* relative to the wild-type strain. (c,d) Same as (a,b) except that the variant can infect individuals who have recovered from infection with the wild type. (e,f) As a function of the variant’s escape capability *e*.

We capture this idea using the *deficiency ratio*, denoted by *d*, which is the ratio between the reproduction number of the mutant strain and that of the wild type, both in a naive population. Hence, *d* = 1 implies no deficiency, which is what has been assumed up to this point, while *d* = 0.5, for example, means that the variant’s reproduction number is half that of the wild type. In Fig 9A and 9B we present the probability of vaccine escape vs. the variant’s deficiency ratio *d* and the mutation rate *μ*, under simultaneous vaccination (Fig 9A) and spatial K = 10 vaccination (Fig 9B). For completeness we consider the range 0.4≤*d*≤1.2; however, as argued above, the middle of this range should be seen as more probable. As expected, in both cases we observe low risk (blue) in the bottom-right corner, either because mutations are rare (small *μ*) or because they are deleterious to the virus (small *d*). In the opposite corner, where *μ* and *d* are large, we observe a high risk (red) under both regimes. The two areas are separated by a band of intermediate risk (green). The crucial point is that under spatial vaccination the low-risk blue area is expanded, while the high-risk red area contracts to only the extreme upper-left corner. This clearly shows that spatial vaccination consistently mitigates the risk of vaccine escape throughout the entire range.

Importantly, it can be seen that the benefit of spatial vaccination, namely how far it shifts the green and red areas to the right, is greater when the variant has higher deficiency (smaller d): while for *d* = 1 the rightward shift is about 0.42 orders of magnitude (×2.6) of *μ*, for *d* = 0.8 it is about 0.56 orders of magnitude (×3.6) of *μ* (these are the respective horizontal distances in between the dotted and solid black curves in Fig 9B). The reason for the difference lies in the variant’s ability to survive before vaccination gives it an advantage. With *d*<<1, the variant has an *R*<<1 before vaccination and in its early stages. Thus, if it appears in these early stages then it will likely not survive. Only variants born late in the vaccination process, when their R exceeds unity, are able to survive, implying that spatial vaccination has a major advantage due to the shortening of the high-risk period. With *d* = 1, in contrast, variants born in the earlier stages have a higher chance of survival–under any vaccination regime–thus reducing the relative advantage of spatial vaccination.

#### A variant that also infects the recovered

We next examine the effect of the variant’s ability to also infect individuals with antibodies from prior infection with the wild type. As expected, the results, for both K = 1 (Fig 9C) and K = 10 (Fig 9D), indicate that such a scenario increases the risk of escape relative to the case in which the variant cannot infect the recovered (Fig 9A and 9B). Note however that even under this scenario, in which the variant also infects the recovered, the spatial vaccination continues to maintain a clear advantage, as can be seen in the enlarged low-risk area (blue) and the diminished high-risk area (red).

#### Escape is partial

Another important issue we examine is *partial* vaccine escape, i.e. the vaccine provides some protection against the escape variant. This scenario appears to fit our current experience with some of the SARS-CoV-2 variants [32,33].

To quantify this scenario, we introduce a parameter measuring the severity of vaccine escape, denoted by *e*. Setting *e* = 1 represents full escape, which is the scenario considered up to this point; *e* = 0.5, for example, means that the vaccine is partially effective against the variant so that the variant escapes it with only 50% probability. In Fig 9E and 9F, we set *d* = 0.7 and examine the impact of introducing *e*<1. It can be seen that the contour lines now have a steep, almost vertical slope in both heat maps. Hence, the risk is largely independent of *e* within the range 0.4≤*e*≤1. Importantly, this risk continues to fall as we shift from simultaneous vaccination (K = 1) to spatial vaccination (K = 10).

#### A combined parameter space

**Fig 10:**
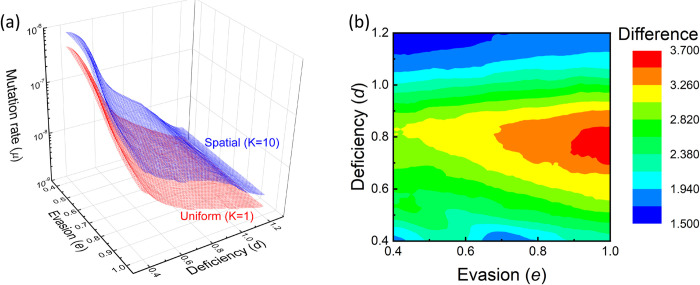
Mutation rate that leads to 50% escape probability (as a function of the variant’s deficiency *d* and escape capability *e*). (a) Spatial vaccination dominates over the entire parameter space. (b) The advantage is maximized for large e and intermediate d, and minimized for low e and extreme d.

To complete the analysis, a combined parameter space is considered in which we vary both the deficiency and escape parameters. Thus, for each combination of *d*∈[0.4,1.2] and *e*∈[0.4,1], the mutation rate *μ* that generates an escape probability of P = 50% is plotted. Fig 10A indeed shows that–over the entire parameter range–spatial vaccination tolerates a higher mutation rate for the same escape probability. Fig 10B investigates how we investigate how the advantage of spatial vaccination depends on the two parameters, by computing the ratio of the above mutation rates. Thus, while a higher rate of evasion increases the advantage of spatial vaccination, that advantage is maximized at intermediate values of the deficiency parameter.

### G. Concluding remarks

#### A uniform global SIR model

The simplified SIR model we present assumes a homogeneous world in which social distancing maintains the reproduction number R at unity and the stream of infections is identical in all countries and regions. Of course, the real world is characterized by a high degree of heterogeneity even across proximate regions [20,21]. Nonetheless, we believe that our qualitative results still hold. The key idea is that a slow and uniform vaccination regime, and in view of the adaptive nature of social distancing measures, the outcome is inevitably a continued high infection rates for an extended period of time. A significant proportion of these infections will occur when vaccination levels are high, implying a high reproduction rate and therefore a high probability of survival for an escape variant. Our analysis, which “mistakenly” uses the average R in each of the regions, ignores the nonlinearity of the mutant’s survival probability in R (which is approximately 1/(1+1/R) for sufficiently large R). However, this effect is small and more importantly, the effectiveness of spatial vaccination–which in each locality dramatically shortens the time during which a high infection level and a high R coexist–remains.

**Fig 11:**
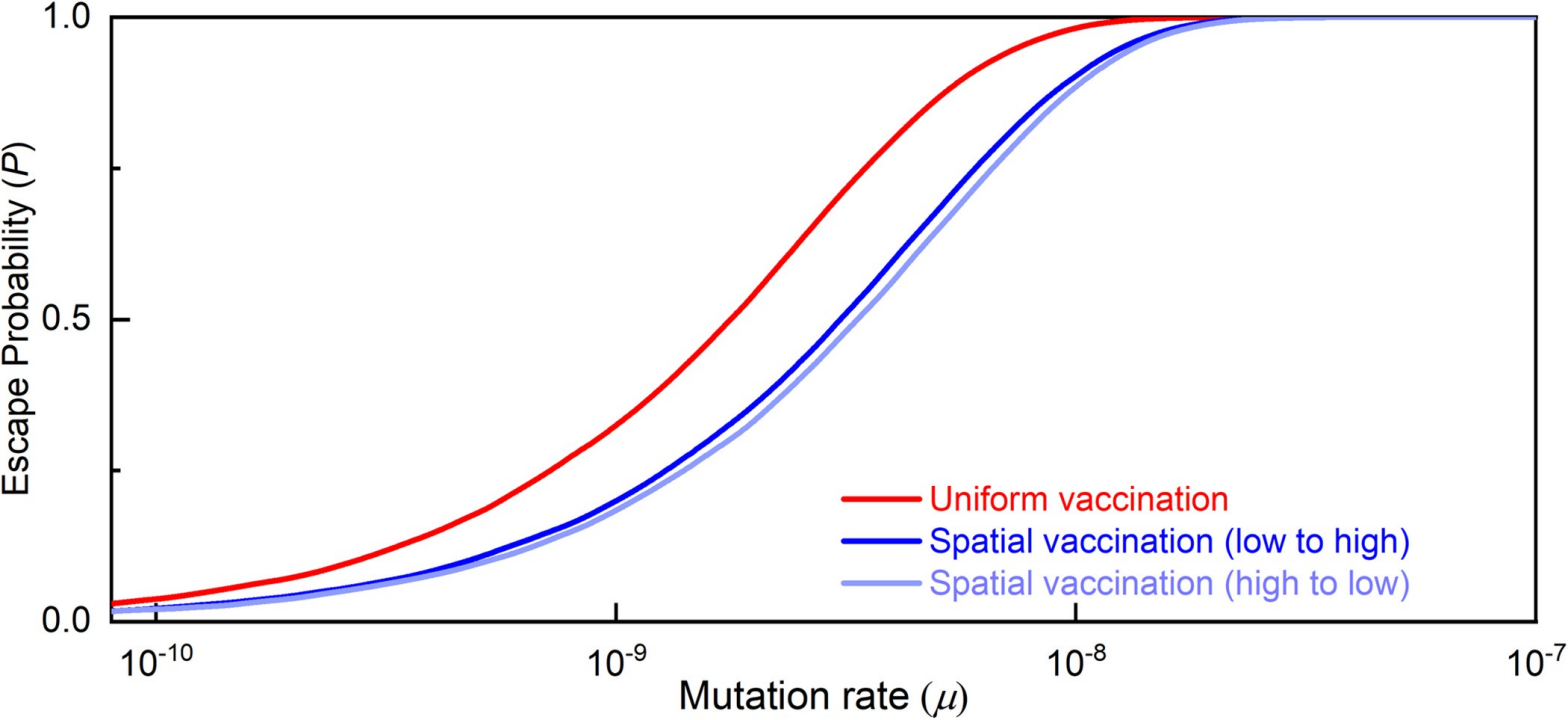
Exploiting heterogeneous infection rates. Even up to a 10-fold difference in infection rates, there is only a slight benefit in vaccinating regions with higher infection rates first.

However, if different localities have different infection rates, this can be exploited by the spatial vaccination strategy. Fig 11 shows a situation in which 10 regions have different infection rates (specifically, region *i*>1 has *i* times more infections than region 1). It can be seen that there is a slight advantage to vaccinating the regions with higher infection rates first. However, optimizing the order of spatial vaccination has a much smaller added benefit than that achieved by spatial vaccination over uniform vaccination.

#### The mutation process

Our model treats the complex process of mutation in a highly simplified manner, assuming that upon each instance of infection, the virus mutates with some probability. In reality, a complex mutation that enables vaccine escape does not take place at the time of infection, but rather it is the result of a combination of single-point mutations occurring within the host, as the virus reproduces within his body. However, this internal process is of little relevance to our analysis since–apart from very rare cases–infections almost always involve a single strain randomly drawn from the cloud of mutations within the infector. Hence, for simplicity we assume that with probability *μ*, a specific host acquires a vaccine-resistant mutation, which is then passed on to other individuals via further infection. We model this hidden process as if the virus mutates upon infection. (Note that the characteristics of the internal process are more important in studying drug resistance, as in [34,35], or in studying partial (one-dose) vaccination which may generate an inter-host fitness advantage for a resistant variant together with a continued increased viral load [36].)

A limitation of our model, however, is that it ignores the possibility of a drift by which a sequence of single-point mutations occurs during the transmissions between individuals, such that only the sum of these mutations generates vaccine escape.

#### The range of the mutation rate and the significance of the reduction in escape risk

While the probability of a single-point mutation in the case of the SARS-COV-2 virus can be computed from its genomic properties, the probability that a combination of mutations generates a variant that is resistant to the current vaccines is unknown at this stage. On the optimistic side, none of the currently prevailing variants fully escapes the current vaccines [37,38], and it appears that most of them are simply improvements of the virus that are to be expected in its adaptation process in the human host. On the pessimistic side, this can be explained by the fact that mass vaccination, which leads to selective pressure for vaccine resistance, has taken place until recently only in countries that account for a small proportion of the world’s population [7,18].

Along this paper, we showed that the type of vaccination regime matters when the mutation rate *μ* is within the range of 10^−10^ to 10^−7^. That is, within that range there is a gap between the best-case instantaneous vaccination benchmark and the worst-case slow vaccination regime that has actually been adopted. We have shown that spatial vaccination eliminates 50% of the excess risk (and even more with the help of the complementary measures described in Section 2C).

Another way to assess the benefit of spatial vaccination is to consider the metric introduced in Section 2A, according to which spatial vaccination permits a higher mutation rate for the same escape risk–by about 0.5 orders of magnitude on average (across possible values of d) before taking into account complementary measures. Translating this number into a probability that escape will be avoided depends on our prior on *μ*, namely what range of values do we view as being reasonable. Lobinska et al. [7] provides a methodology to compute an upper bound based on the fact that a vaccine-resistant variant has not appeared until now in the highly vaccinated countries (which currently account for 5–10% of world population) and arrives at an estimate of about 10^−6^. However, without a lower bound it is hard to justify employing the spatial strategy. Such a lower bound will be generated if we encounter the adverse scenario that a resistant variant emerges during the first round of global vaccination (and the lower bound will be stricter the earlier the variant is encountered). If this leads to a second round of vaccination with an updated vaccine, then the spatial strategy might be considered. Moreover, the combination of the lower and upper bounds may provide us with a fairly narrow range, which will justify the use of spatial vaccination.

## 3. Methods

### SIR model with regions and mutations

A world with population *N* is naturally divided into *K* identical regions *k* = 1…K with little interaction between them. Let 0<*c*<<1 (the contact ratio) denote the proportion of a person’s interactions that are with people living outside of her own region. The dynamics of the wild-type infection in each region follows a deterministic SIR model, with interactions between the regions. We set the recovery rate *r* to 1 so that time is measured in infection cycles (about 4 days in the COVID-19 case) and for simplicity set the death rate to 0. The wild type’s basic reproduction rate *R*_0_ is set to 4, which is about that of the current dominant strains, so that the infection rate is *β*_0_ = *rR*_0_ = 4. We assume that social distancing measures–multiplicative factors *l*_*k*_−are set such that the rate of infection in each region does not exceed an acceptable proportion *h* of the population (per 4-day infection cycle).

We augment the model with a vaccination process *v*_*k*_(*t*) that starts at time *t*_0_ (= one year after the pandemic’s start) and is subject to an aggregate capacity constraint *Σv*_*k*_(*t*)≤*v* such that 80% of the population can be vaccinated within one year (i.e., *v* = 80%·4/365 is the proportion vaccinated per infection cycle). Both the susceptible and the recovered are vaccinated. Under uniform vaccination, *v*_*k*_(*t*) = *v*, i.e. a proportion v of every region’s population is vaccinated per infection cycle. Under spatial vaccination, *v*_*k*_(*t*) = *v*·*K* for *t*_*k*−1_≤*t*≤*t*_*k*_, where *t*_*k*_ is the point in time at which 80% of region *k*’s population is vaccinated; otherwise *v*_*k*_(*t*) = 0. Thus, when it is region *k*’s turn, it is vaccinated at a *K*-fold faster rate than under uniform vaccination. Under “mixed” vaccination, we start at time *t*_0_ with the uniform regime until 15% of the population in each region is vaccinated and then switch to the spatial regime.

Each wild-type infection has a probability *μ* of turning into a vaccine-resistant variant (“mutant”). While the wild type cannot infect vaccinated individuals, the mutant can. However, it suffers a fitness deficiency such that its basic reproduction number $R_{0}^{m}$ equals *d*·*R*_0_ (while we typically expect *d* to be lower than 1, we also analyze the case in which it is above 1). If a new mutant occurs, we track the (discrete) number of infections using a random walk process until it either dies out or succeeds in generating a large number of infections (and then becomes dominant). The parameter *e*≤1 captures the degree of the variant’s escape from the vaccine (1 means full escape; 0 means no escape). We also consider two cases: that the resistant variant can or cannot infect individuals who have recovered.

**Fig 12:**
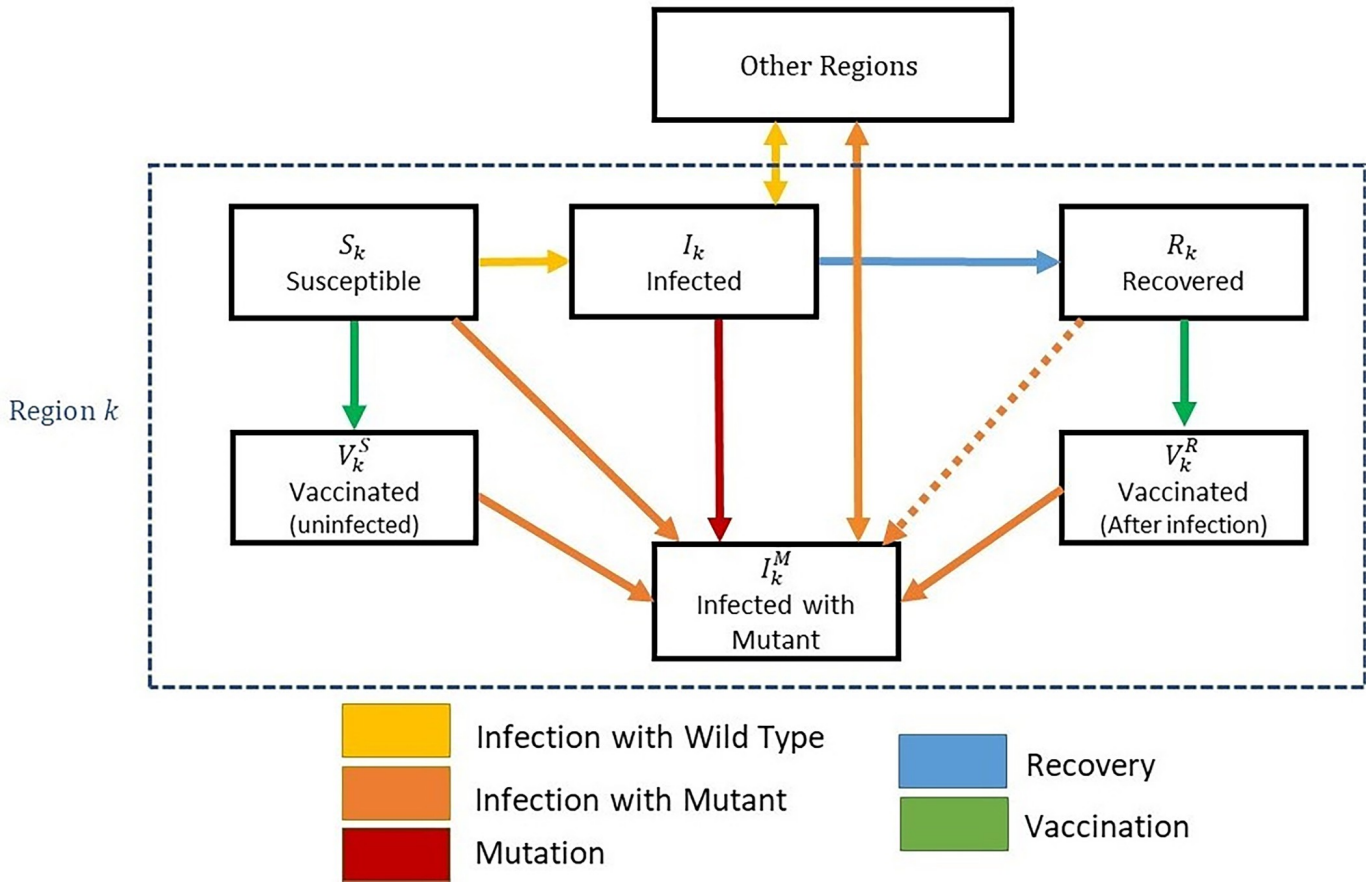
The flows between the different states–within a given region k and in between regions.

The different states of the augmented SIR model and the transition flows between different states are shown in Fig 12:

**The deterministic SIR equations for the wild**-**type strain** in each region *k* are as follows (all state variables are defined as proportions of the population, i.e. between 0 and 1):

W1. Infected: ${İ}_{k}=l_{k}\beta_{0}S_{k}{\hat{I}}_{k}(1-\mu )-{rI}_{k}$
W2.
  where ${\hat{I}}_{k}=(1-c)I_{k}+\frac{c}{K-1}\Sigma_{j\neq k}I_{j}$ is the effective infection pool for region *k*,
  and where $l_{k}=min\left(1,\frac{h}{\beta_{0}S_{k}{\hat{I}}_{k}(1-\mu )}\right)$ so that ${İ}_{k}=min\left(\beta_{0}S_{k}{\hat{I}}_{k}(1-\mu ),h\right)-{rI}_{k}$
W3. Susceptible: ${Ṡ}_{k}=-l_{k}\beta_{0}S_{k}{\hat{I}}_{k}(1-\mu )-v_{k}\frac{S_{k}}{S_{k}+R_{k}}$
W4. Recovered: ${Ṙ}_{k}={rI}_{k}-v_{k}\frac{S_{k}}{S_{k}+R_{k}}$
W5. Vaccinated (and were not infected earlier): ${\dot{V}}_{k}^{S}=v_{k}\frac{S_{k}}{S_{k}+R_{k}}$
W6. Vaccinated (and were infected and recovered earlier): ${\dot{V}}_{k}^{R}=v_{k}\frac{R_{k}}{S_{k}+R_{k}}$
W7. The initial conditions are: $I_{k}=h,\;S_{k}=1-h,\;R_{k}=V_{k}^{S}=V_{k}^{R}=0$.

(Note that the separation between the two types of vaccinated individuals is needed only for the case in which the resistant variant cannot infect people who have already recovered from the wild type (see Case 1 below). In this case it can infect only those in V^S^. If the variant is resistant to wild-type antibodies, i.e. it also infects the recovered (Case 2), then we can merge V^S^ and V^R^ into one group V.)

*The process for the resistant strain is discrete*. Initially, the (absolute) number $I_{k}^{m}$ of mutant infections in region *k* is 0. Each $I_{k}^{m}$ evolves according to a random walk, dictated by three Poisson processes: (1) arrival of new mutations; (2) infections by the resistant variant; and (3) recoveries. The arrival rate of these processes are given by:

M1. Mutation ($I_{k}^{m}$ increased by 1): $\mu \cdot l_{k}\beta_{0}S_{k}{\hat{I}}_{k}\cdot \frac{N}{K}$
  (Note that for not yet vaccinated regions his is simply $\mu \cdot h\cdot \frac{N}{K}$.)
M2. Infections ($I_{k}^{m}$ increased by 1):
  ○ Case 1 (variant does not infect the recovered): $l_{k}\beta_{0}^{m}(S_{k}+{eV}_{k}^{S})\hat{I_{k}^{m}}$
  ○ Case 2 (variant infects the recovered): $l_{k}\beta_{0}^{m}(S_{k}+{e(V}_{k}^{S}+V_{k}^{R}+R_{k}))\hat{I_{k}^{m}}$
  where $\beta_{0}^{m}$ equals *d*·*β*_0_ and where $\hat{I_{k}^{m}}=(1-c)I_{k}^{m}+\frac{c}{K-1}\Sigma_{j\neq k}I_{j}^{m}$ is the effective infection pool for region *k*.
  (For simplicity we assume that the variant cannot infect people currently infected by the wild type.)
M3. Recoveries ($I_{k}^{m}$ decreased by 1): $r\cdot I_{k}^{m}$

### Definition of the outcome (of one iteration of the simulation)

- We say that the pandemic is over and the variant has died out when *Σ*_*k*_*I*_*k*_<1/*N* and $\Sigma_{k}I_{k}^{m}=0$.
- We say that the variant has taken over when $\Sigma_{k}I_{k}^{m}$ grows to beyond 30.

(Note that, since mutations are rare, infections can reach 30 only if the reproduction number is much larger than 1. Moreover, since the lockdown is further eased as vaccinations proceed, the mutation’s effective reproduction number can only grow. Thus, the path from 30 to taking over is almost guaranteed. Indeed, simulations show that setting a higher threshold does not result in a lower probability of reaching it.)

**The complementary measures** (see Section 2C) are modeled using the following modifications:

- Travel restrictions: Let *c*^−^<*c* denote the reduced interaction rate between regions with different vaccination statuses and denote the region being currently vaccinated by k*. We change the definition of ${\hat{I}}_{k}$ as follows:
- ${\hat{I}}_{k}=(1-\frac{(k^{*}-2)c+({K-k}^{*}+1)c^{-}}{K-1})I_{k}+\frac{c}{K-1}\Sigma_{j<k^{*},j\neq k}I_{j}+\frac{c^{-}}{K-1}\Sigma_{j\geq k^{*}}I_{j}$ if *k*<k*
  ${\hat{I}}_{k}=(1-c^{-})I_{k}+\frac{c^{-}}{K-1}\Sigma_{j\neq k}I_{j}$ if *k* = k*
  ${\hat{I}}_{k}=(1-\frac{k^{*}c^{-}+({K-k}^{*}-1)c}{K-1})I_{k}+\frac{c^{-}}{K-1}\Sigma_{j\leq k^{*}}I_{j}+\frac{c}{K-1}\Sigma_{j>k^{*},j\neq k}I_{j}$ if *k*>k*
  and correspondingly the definition of $\hat{I_{k}^{m}}$.
- Contact tracing for the resistant strain: In regions that have already been vaccinated and in which the total number of wild-type infections has been significantly reduced, i.e. ${İ}_{k}<0.1h$, we further multiply the resistant strain’s infection rates (cases 1 and 2 in M2 above) by *l*^*CT*^ = 0.5.
- A moving temporary lockdown: For the region k* being vaccinated, we replace the definition of *l*_*k*_ (W1 above) with $l_{k^{*}}=min\left(0.8,0.8\frac{1}{\beta_{0}S_{k}}\right)$ which implies that the reproduction number is pushed down to 0.8 rather than to 1.
