# Correction: A spatial vaccination strategy to reduce the risk of vaccine-resistant variants

**DOI:** 10.1371/journal.pcbi.1011608

**Published:** 2023-10-30

**Authors:** Xiyun Zhang, Gabriela Lobinska, Michal Feldman, Eddie Dekel, Martin A. Nowak, Yitzhak Pilpel, Yonatan Pauzner, Baruch Barzel, Ady Pauzner

The Funding statement is incomplete. The complete statement is: X.Z. was supported by the NNSF of China under Grant No. 12105117, the Fundamental Research Funds for the Central Universities (Grant No. 21621007), Guangdong Basic and Applied Basic Research Foundation (Grant No. 2022A1515010523), the Science and Technology Planning Project of Guangzhou (Grant No. 202201010360), and E.D. was supported by NSF grant SES-1919494. A.P. was supported by the Pinhas Sapir Center for Development and the Foerder Institute for Economics Research. The funders had no role in study design, data collection and analysis, decision to publish, or preparation of the manuscript.

## References

[1] Zhang X, Lobinska G, Feldman M, Dekel E, Nowak MA, Pilpel Y, et al. (2022) A spatial vaccination strategy to reduce the risk of vaccine-resistant variants. PLoS Comput Biol 18(8): e1010391. 10.1371/journal.pcbi.1010391 35947602 PMC9394842

